# Functionally Heterogenous Macrophage Subsets in the Pathogenesis of Giant Cell Arteritis: Novel Targets for Disease Monitoring and Treatment

**DOI:** 10.3390/jcm10214958

**Published:** 2021-10-26

**Authors:** Idil Esen, William F. Jiemy, Yannick van Sleen, Kornelis S.M. van der Geest, Maria Sandovici, Peter Heeringa, Annemieke M. H. Boots, Elisabeth Brouwer

**Affiliations:** 1 Department of Rheumatology and Clinical Immunology, University of Groningen, University Medical Center Groningen, 9700 RB Groningen, The Netherlands; w.f.jiemy@umcg.nl (W.F.J.); y.van.sleen@umcg.nl (Y.v.S.); k.s.m.van.der.geest@umcg.nl (K.S.M.v.d.G.); m.sandovici01@umcg.nl (M.S.); m.boots@umcg.nl (A.M.H.B.); e.brouwer@umcg.nl (E.B.); 2 Department of Pathology and Medical Biology, University of Groningen, University Medical Center Groningen, 9713 GZ Groningen, The Netherlands; p.heeringa@umcg.nl

**Keywords:** giant cell arteritis, macrophages, imaging, biomarkers

## Abstract

Giant cell arteritis (GCA) is a granulomatous large-vessel vasculitis that affects adults above 50 years of age. In GCA, circulating monocytes are recruited to the inflamed arteries. With cues from the vascular microenvironment, they differentiate into macrophages and play important roles in the pathogenesis of GCA via pro-inflammatory cytokine production and vascular remodeling. However, a deeper understanding of macrophage heterogeneity in GCA pathogenesis is needed to assist the development of novel diagnostic tools and targeted therapies. Here, we review the current knowledge on macrophage heterogeneity and diverse functions of macrophage subsets in the pathogenesis of GCA. We next discuss the possibility to exploit their heterogeneity as a source of novel biomarkers and as targets for nuclear imaging. Finally, we discuss novel macrophage-targeted therapies and future directions for targeting these cells in GCA.

## 1. Introduction

Giant cell arteritis (GCA) is an auto-inflammatory disease that affects medium- and large-sized vessels in adults older than 50 years. GCA belongs to a disease spectrum that includes cranial GCA (C-GCA) and large-vessel GCA (LV-GCA), with or without overlapping polymyalgia rheumatica (PMR). The vasculitis in C-GCA mainly affects cranial arteries leading to symptoms such as headache, jaw claudication and vision loss [1]. Large-vessel (LV-GCA) GCA affects the aorta and its major branches leading mostly to symptoms such as weight loss, fatigue, night sweats and fever. Inflammation and remodeling in GCA can cause ischemic complications or aortic aneurysms and dissection [2]. C-GCA and LV-GCA may be often present together. Indeed, imaging studies reported a high percentage (up to 83%) of overlapping C-GCA and LV-GCA [3,4,5,6]. The American College of Rheumatology (ACR) 1990 classification criteria for GCA are exclusively based on the assessment of cranial features of GCA in addition to the age of the patient and an elevated erythrocyte sedimentation rate (ESR) and a positive temporal artery biopsy (TAB) [7]. However, there is room for improvement in the diagnosis of GCA. Even though a TAB has a high specificity, it is invasive with limited sensitivity. More recently, the European League Against Rheumatism (EULAR) proposed an expansion of the original classification criteria recommending the inclusion of clinical features of LV-GCA and PMR as well as the use of molecular imaging (ultrasound or magnetic resonance imaging (MRI), computerized tomography (CT), positron-emission-tomography (PET)-CT) [8,9]. However, the availability of imaging tools for GCA diagnosis remains challenging in daily care [10,11]. Therefore, reliable and disease-specific biomarkers are highly needed for GCA.

To date, glucocorticoids (GCs) are the mainstay of treatment for GCA. However, long-term GC usage causes serious adverse events such as the development of type II diabetes, hypertension, osteoporosis and increased risk of infection [12,13]. Additionally, the majority of patients experience relapses while on GCs treatment [14]. Although the acute-phase response is suppressed by GCs, there is proof of ongoing inflammation in the affected vascular tissues [15,16,17]. Therefore, alternative, more effective treatment options are highly needed in newly diagnosed and relapsing patients. Methotrexate has long been used with some GC-sparing effect in GCA [18]. More recently, the interleukin 6 receptor blocker, Tocilizumab, was shown to induce sustained GC-free remission in around 50% of the GCA patients after 52 weeks of treatment [19]. However, after 52 weeks of tocilizumab treatment, magnetic resonance angiography (MRA) revealed that vascular inflammation continues or reappears in two-third of GCA patients despite clinical remission [20]. Furthermore, an investigation of biomarkers in tocilizumab-treated patients documents immunological signs of subclinical disease activity in GCA patients especially in the early stages of treatment emphasizing the need for long-term treatment with tocilizumab [21]. Thus, these results pinpoint that current treatments do not abolish the local inflammation efficiently. As the persistent inflammation in the vessel wall is largely granulomatous [15], targeting macrophages may improve the effectiveness of immunosuppression and may induce lasting remission. Although the immunopathogenesis of GCA is unknown, it is most likely determined by the interaction of multiple factors including genetic susceptibility, environmental factors and aging of the immune system. Several studies showed an association between HLA-DRB1*04 and more recently also HLA-B*15:01 alleles with GCA, thereby suggesting the contribution of both MHC class II and class I genes to genetic susceptibility in GCA development [22,23]. Additionally, the role of activated dendritic cells (DCs), T cells, macrophages and type I IFN-related pathways in the pathogenesis of GCA points to a relationship between infection and the initiation of GCA [24]. However, the assessment of the microbiome in GCA-affected arteries has provided inconclusive and conflicting results leaving the question on the involvement of pathogens in the pathogenesis of GCA unresolved [25,26,27]. Besides, the aging of the immune system may also contribute to increased susceptibility to infections and, in combination with vascular ageing, accelerate the vasculopathy of GCA [24].

**Figure 1:**
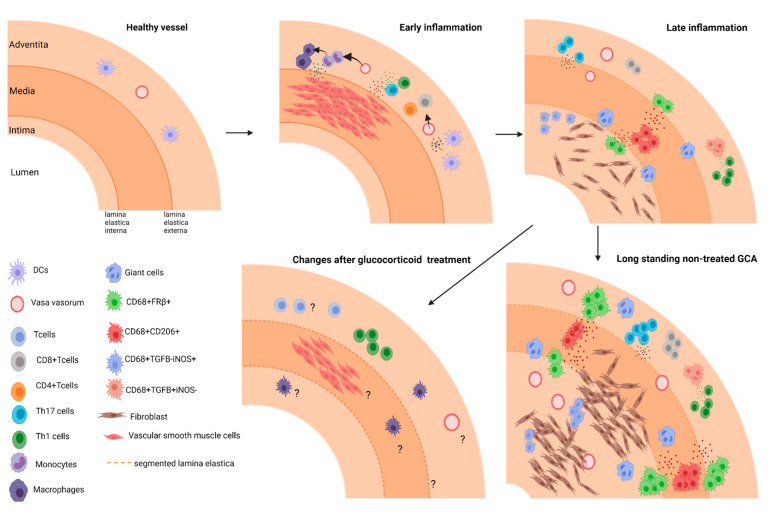
**Model of different stages in arterial inflammation in GCA. Early inflammation:** Dendritic cell activation recruits T cells (CD4+ and CD8+) to the vessel wall and drives activation, and polarization of T cells. Secreted soluble factors lead to monocyte recruitment and their differentiation into macrophages. **Late inflammation:** Different macrophages subsets skewed by environmental cues contribute to tissue remodeling in GCA. CD206+ GM-CSF skewed macrophages producing MMPs mediate tissue destruction; FRβ+M-CSF skewed macrophages and fibroblast are activated and drive intimal proliferation. **Long standing GCA:** ultimately inflammation leads to vascular occlusion. **Changes after glucocorticoid treatment:** The effect of treatment on cellular infiltrates and vascular lesions/repair remain to be elucidated.

It is well-known that macrophages play critical roles in the pathogenesis of GCA, as they mediate inflammatory responses affecting processes like tissue remodeling and angiogenesis (Figure 1) [28]. Moreover, some of the macrophages in the vessel-wall fuse and form multinucleated giant cells, which is a hallmark of GCA [29]. Treatment with GCs cannot sufficiently suppress local inflammation which likely leads to relapse (Figure 1) [15]. Macrophages are highly plastic cells that can rapidly change their phenotypes upon cues from the tissue microenvironment. Recent research showed that a more comprehensive examination is needed for the characterization of macrophage heterogeneity both in phenotype and function in tissues [30,31,32]. Macrophage subsets involved in the vasculopathy of GCA could be exploited for diagnostic purposes, as a source of biomarkers, as targets for imaging and as a targets for treatment. In this review, we will discuss the current knowledge on the mechanisms underlying the distinct macrophage phenotypes and functions involved in the pathogenesis of GCA and the implications for improved diagnosis (biomarkers, imaging), monitoring (biomarkers, imaging), prognosis (biomarkers) and therapy in GCA.

### 1.1. Pathogenesis of GCA

The initiation of the inflammatory response in the arterial vessel wall of GCA patients is not well understood. It is suggested that vascular dendritic cells are activated through Toll-like receptors (TLR) via unknown endogenous or exogenous ligands, leading to the production of chemokines (CCL18, CCL19, CCL20 and CCL21) and cytokines (IL-1, IL-6, IL-12, IL-18 and IL-33). These cytokines and chemokines recruit CD4+ T cells to the arterial wall and polarize them toward Th1 and Th17 cells. The infiltrating Th1 and Th17 cells produce proinflammatory cytokines including IFN-γ and IL-17, respectively. IFN-γ activates macrophages towards a proinflammatory phenotype that produces various proinflammatory cytokines and chemokines. Additionally, IFN-γ induces vascular endothelial growth factor (VEGF) and chemokine (CCL2, CXCL9, CXCL10 and CXCL11) expression by vascular smooth muscle cells (VSMCs) and endothelial cells (ECs) leading to neoangiogenesis and recruitment of more monocytes and T cells to the site of inflammation [33] (Figure 1).

Studies on affected vessels of GCA patients point to a central role of macrophages in the vasculopathy. Once recruited, monocytes differentiate into macrophages and produce the pro-inflammatory cytokines IL-6, IL-1β and TNF-α that amplify the inflammatory response. These cytokines, particularly IL-6, initiate a systemic response of the body characterized by high levels of acute-phase markers in the blood giving rise to the systemic symptoms of GCA, such as fever, weight loss and malaise. In addition, macrophages at the site of inflammation play a critical role in vascular remodeling by promoting angiogenesis, intimal hyperplasia and tissue destruction. Macrophages are capable instigators of neoangiogenesis by the secretion of VEGF, IL-33 and YKL-40. Platelet-derived growth factor (PDGF), which is produced by macrophages, promotes VSMC and fibroblast migration and proliferation leading to intimal hyperplasia and eventually vessel-wall occlusion. (Figure 1) Furthermore, activated macrophages are the main contributors to tissue destruction by producing matrix metalloproteinases (MMPs) [33,34,35]. Therefore, gaining a deeper understanding of monocyte and macrophage involvement in GCA pathogenesis is vital.

### 1.2. Monocytes in GCA

Monocytes are the precursors of tissue macrophages and in the blood of GCA patients, altered dynamics and distribution of monocyte subsets have been documented [17]. At diagnosis, GCA patients present with elevated counts of circulating monocytes, and these elevated counts were found to associate with C reactive protein (CRP) levels [17]. During treatment with glucocorticoids, and even after termination of treatment, monocyte counts remained elevated in GCA patients. Monocytes display heterogeneity in their phenotype and function, and are currently subdivided into three subsets: classical monocytes (CD14^bright^CD16^neg^), intermediate monocytes (CD14^bright^CD16+) and non-classical monocytes (CD14^dim^CD16+). CD16+ monocytes were shown to be increased with age and associated with different inflammatory diseases such as systemic lupus erythematosus (SLE), rheumatoid arthritis (RA) and anti-neutrophil cytoplasmic antibody (ANCA)-associated vasculitis (AAV) [36,37]. The monocytosis observed in GCA patients was attributed to an expansion of the classical monocyte subset, although elevated intermediate monocyte counts have also been described [37,38]. Even though total monocyte counts remained high during treatment with glucocorticoids, substantially lower non-classical monocyte counts were noted, likely due to enhanced induction of apoptosis [37,39].

Alterations in the functioning and migration of monocytes of GCA patients could play a role in initiating and fueling vascular inflammation. Monocyte subsets use different chemotaxis pathways to enter tissues, as classical monocytes mainly depend on the CCR2-CCL2 axis, and non-classical monocytes depend on the CX3CR1-CX3CL1 axis [40]. Further research on CCL2-CCR2 and CX3CR1-CX3CL1 pathways in tissue demonstrated that the majority of macrophages in TABs of GCA patients resemble non-classical monocytes with CD16 and CX3CR1 expression, but often lack CCR2 expression [37]. Albeit to a lower extent, influx of CCR2 expressing macrophages resembling the classical monocyte phenotype were also detected in the vessel wall of GCA patients [37,40]. Elevated CCL2 expression by VSMCs caused by the inflammatory microenvironment has been reported in GCA [40]. These reports suggest a central role for both classical and non-classical monocytes in the vasculopathy of GCA. In addition, tissue migrated monocytes/macrophages may aid the migration of T-cells to the vessel wall as well, through their production of MMP-9, which breaks down the extracellular matrix [41]. Further evidence indicates that the non-classical monocyte subset is the main source of MMP-9 and associated enzymes, in addition to pro-angiogenic factors such as YKL-40 [35,42,43]. Moreover, (classical) monocytes of GCA patients show upregulated CD64 expression but lowered expression of folate receptor β, which is likely a sign of an activated phenotype [41,44].

### 1.3. Macrophage Heterogeneity and Their Roles in the Vasculopathy of GCA

Upon migration of circulating monocytes to vascular tissue lesions, monocytes differentiate into macrophages. Although it is known that macrophages are highly plastic cells with the ability to adapt to the microenvironment, the knowledge regarding the phenotypic and functional diversity of macrophages in GCA tissues is steadily growing. Before, macrophages were classified into two major subtypes, widely known as the M1 and M2 macrophages. M1 type macrophages are induced by IFN-γ, and considered pro-inflammatory due to their capacity to produce proinflammatory cytokines (such as IL-1β, IL-6, IL-12 and IL-23), growth factors (VEGF, PDGFs), MMPs and reactive oxygen species (ROS). These factors play an important role in the immunopathology of GCA [45,46]. In contrast to M1 macrophages, M2 macrophages are defined as an anti-inflammatory and tissue repairing subtype. They are the source of IL-10 and express the macrophage mannose receptor (CD206) [45,46,47,48]. However, the distinction between M1 and M2 macrophage subsets is largely based on in vitro experiments under controlled conditions which is now regarded an oversimplification of the much more complex environment in tissues. Indeed, dedicated tissue studies have shown macrophage phenotypes with mixed traits of both M1 (CD64 expression) and M2 (CD206/FRβ) macrophages in pathological conditions including GCA [32,49,50,51,52].

A variety of important soluble mediators have been identified in GCA such as IFN-γ, PDGF, IL-17, IL-6 and GM-CSF that are secreted by different cell types in the vessel wall microenvironment and can modulate macrophage heterogeneity. As macrophages are highly plastic, it is no surprise that different phenotypes of macrophages with different functions may be present in GCA-affected vessel-walls. This notion of macrophage heterogeneity was first reported by Weyand et al. [53]. Immunohistochemical analysis revealed a functional heterogeneity of tissue-infiltrating macrophages where TGF-β1(+)iNOS (−) macrophages, localized in the adventitia in the vicinity of IFN(+) CD4+ T cells, contribute to IL-1β and IL-6 production. On the other hand, TGF-β1(−)iNOS(+) macrophages were found in the intimal layer of the inflamed artery and expressed MMP-2, a collagenase involved in tissue destruction [53,54].

Recently, our group reported a distinct spatial distribution of CD206+/YKL-40+/MMP-9+ macrophages and FRβ+/CD206- macrophages linked to tissue destruction and intimal hyperplasia, respectively, in GCA [35]. The observed macrophage phenotypic heterogeneity is likely caused by the local production of GM-CSF and M-CSF. The ability of GM-CSF and M-CSF to induce overexpression of CD206 and FRβ, respectively, has been reported previously [55,56,57]. CD206+YKL-40+MMP-9+ macrophages located in the media and media borders along the sites of elastic lamina degradation are likely skewed by local GM-CSF signals. Indeed, previous reports also revealed that MMP-2 and MMP-9 were expressed by macrophages and giant cells adjacent to the internal elastic lamina [58,59]. Both MMP-2 and MMP-9 play roles in the pathogenesis of GCA due to their ability to degrade elastin [41,60]. The overexpression of MMP-9 by macrophages in GCA vessels may be governed by YKL-40, acting as an upstream signal. YKL-40 also has the proangiogenic ability to induce vasa vasorum formation. Overall, these results showed that the spatial distribution of CD206+/YKL-40+/MMP-9+ macrophages in the media and media borders where elastic lamina degradation takes place matches with a tissue destructive and proangiogenic role of these macrophages.

In contrast to CD206+/MMP-9+/YKL-40+ macrophages, FRβ+ macrophages were mainly found in the adventitia and inner-intima, adjacent to CD206+MMP-9+ macrophages [44,61]. FRβ+ macrophages are likely skewed by local M-CSF signals as opposed to GM-CSF signals that have been reported to diminish FRβ expression by macrophages [55,56]. In inflamed GCA vessels, M-CSF expression was found to be highly localized in the area with CD206+/MMP-9+/YKL-40+ macrophages, suggesting that M-CSF produced by CD206+/MMP-9+/YKL-40+ macrophages primes adjacent macrophages to express FRβ. Intriguingly, significantly higher numbers of infiltrating FRβ+ macrophages were detected in the inner intima of vessels with massive intimal hyperplasia, suggesting their importance in promoting fibroblast proliferation. Indeed, M-CSF-primed macrophages, rather than GM-CSF-primed macrophages, secreted high levels of platelet-derived growth factor (PDGF)-AA, a factor known to promote fibroblast migration and proliferation. These data imply that spatial gradients of GM-CSF and M-CSF in the inflamed vessel wall might be responsible for the distinct macrophage subset distribution. Thus, these studies underline that the microenvironment shapes the phenotype and function of macrophages in the vasculopathy of GCA.

Th1 and Th17 polarized cells in the inflamed arteries also impact the phenotype and function of local macrophages. Th1 cells release IFN-γ, which primes macrophages to be more pro-inflammatory. IFN-γ activated macrophages produce PDGF. PDGF leads to activation, and proliferation of VSMCs, that subsequently migrate to the intima of the vessels, obstructing the lumen. Furthermore, the Th17 cytokine IL-17 induces macrophages to produce pro-inflammatory cytokines characteristic for “M1” like macrophages, but at the same time also stimulates CD163 expression, indicative for “M2” like polarization [62]. Moreover, T cells were identified as an essential source of GM-CSF in GCA TABs which may skew macrophages towards a CD206+ phenotypes in GCA lesions [63].

**Figure 2:**
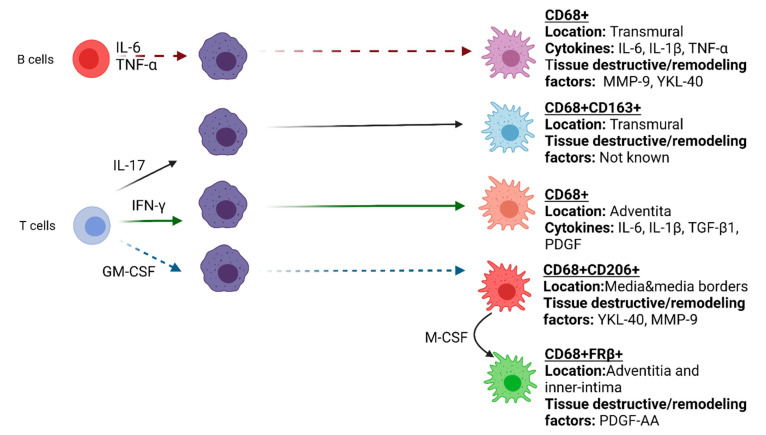
Functional heterogeneity of macrophages in GCA affected vessel-wall.

Recent findings regarding IL-6-producing B cells in GCA indicate a potential role of B cells in GCA pathogenesis. B cells have been described in the adventitia and media of GCA temporal artery and aorta, mostly colocalized with infiltrated CD3+ T cells [64]. Such lymphoid aggregates that show compartmentalization of T and B cells were defined as artery tertiary lymphoid organs (ATLOs) [65,66]. Investigation of ATLOs in GCA demonstrated an association with the ectopic expression of CXCL13 and B-cell activating factor (BAFF) which were shown to increase following in vitro stimulation of temporal arteries with IL-6 [66]. In vitro investigation on the interaction between B cells from GCA and macrophages revealed that soluble factors secreted by B cells enhanced pro-inflammatory cytokine production (IL-6, IL-1β andTNF-α) and induced higher expression of tissue-destructive factors (MMP-9, YKL-40) in macrophages [67]. Furthermore, GM-CSF producing B-cells that can efficiently activate myeloid cells were identified in multiple sclerosis, another autoimmune disease [68,69]. This suggests an interesting interaction between B-cells and macrophages, a notion that requires additional investigation in GCA. Overall, in GCA, different cell types and microenvironments direct functional and phenotypical diversity of macrophages (Figure 2). Novel high dimensional proteomic and transcriptomic methods such as GeoMX^TM^ (Nanostring) and Visium Spatial gene expression platform (10× Genomics) may aid in identifying the macrophage phenotypes in GCA lesions in more detail and provide better insight on macrophage heterogeneity and their specific functions in the vasculopathy of GCA.

### 1.4. Macrophage Metabolism in GCA

Activation of different metabolic pathways has been linked to different phenotypes of immune cells. Our increased knowledge on immune cell metabolism advocates its contribution to the pathogenesis of chronic inflammatory diseases. Therefore, understanding the metabolic pathways of macrophages at the cellular level is critical in elucidating their specific roles in the pathogenesis of chronic inflammatory diseases.

Environmental stimuli steer macrophages towards different metabolic pathways. The metabolism of “M1” (IFNγ-skewed) activated macrophages is characterized by a high rate of aerobic glycolysis, called Warburg metabolism, and impaired oxidative phosphorylation (OXPHOS). On the other hand, OXPHOS and fatty acid oxidation (FAO) are enhanced in “M2” macrophages after stimulation with IL-4 and IL-13 [70,71,72]. Possibly, activated macrophages skewed by LPS/IFNγ utilize glycolysis for rapid, short-term activation that is needed at the site of infection/inflammation, whereas IL-4-induced macrophages rely on OXPHOS that provides better energy support for prolonged cell survival and for prolonged responses to battle parasitic infections [72]. Additionally, it was shown that after macrophage polarization, reprogramming of metabolic pathways contributes to critical functional changes in macrophages [72,73,74].

Activated macrophages stimulated and skewed by IFNγ express iNOS to produce nitrous oxide (NO) from arginine and to form ROS [72]. Additionally, in activated macrophages, the Tricarboxylic acid (TCA) cycle (Krebs cycle) is broken leading to citrate and succinate accumulation. Citrate accumulation carries an important role in the production of NO, ROS and prostaglandins, thereby contributing to a pro-inflammatory and tissue-destructive macrophage phenotype [75,76]. iNOS(+) macrophages were detected in the TABs of GCA patients, thereby demonstrating the presence of metabolically active cells in the inflamed vessel. Besides citrate, succinate accumulation in activated macrophages results in HIF-1α activation that induces inflammatory mediators such as IL-1β [77]. IL-1β has been described as one of the important factors contributing to the pathogenesis of GCA [44,78]. The regulation of IL-1β via metabolic reprogramming in macrophages points to a possibility of metabolic activation via HIF-1α in GCA. Besides the pro-and anti-inflammatory stimulants, mitochondrial products could aid the identification of different subsets of macrophages. Gene expression analysis and immunohistochemical staining of inflamed and non-inflamed TABs revealed an overlap between mitochondrial activation and MMP-2 expression. Thus, the production of ROS and MMP-2 may distinguish a macrophage subset likely involved in tissue destruction [79].

More recently, our group studied the roles of GM-CSF and M-CSF in skewing different macrophage populations in GCA. Reports have shown that stimulation of macrophages with M-CSF, demonstrated increased expression of both glycolytic and TCA cycle enzymes resulting in increased glycolysis and OXPHOS [73,80]. On the other hand, stimulation of macrophages by GM-CSF affects glucose and lipid metabolism. Inhibition of LPS-stimulated glycolysis by 2-deoxyglucose decreased GM-CSF-mediated TNF-α, IL-1β and IL-6 production levels [81]. All in all, these data suggest possible alteration of metabolic activities in the macrophages found in GCA affected vessels which may be exploited to target specific macrophage populations for diagnostics and therapeutic purposes.

### 1.5. Aging Macrophages

GCA is a disease that exclusively affects people over 50 years of age. With aging, systemic low-grade inflammation increases which is activated by damage associated molecular patterns (DAMPs) in the absence of acute inflammatory stimuli and/or pathogen-associated molecular patterns (PAMPs). This constant low-grade inflammation drives tissue and organ damage over time and was coined inflammaging [49,82,83,84,85]. Thus, the inflammaging process and the involvement of cellular aging monocytes and macrophages in the pathogenesis of GCA cannot be ignored.

Cellular senescence is an essential process in aging. The number of senescent cells at the pathologic sites in chronic diseases increases with age [86,87]. Senescent cells secrete pro-inflammatory cytokines, chemokines and tissue-destructive proteins referred to as the senescence-associated secretory phenotype (SASP). The SASP is also able to induce senescence of the surrounding healthy cells [86]. Cellular senescence has not been assessed in vascular tissues of GCA patients; however, the literature indicates its possible role in GCA pathology. Counts of aged non-classical CD14^dim^CD16+ monocytes significantly increase with age. Non-classical monocytes have short telomeres, demonstrate a distinct pro-inflammatory phenotype and have characteristics of senescent cells [88,89]. Interestingly, non-classical monocyte proportions were found decreased in the circulation of GCA patients [37,90]. The relative decrease of non-classical monocytes in GCA may imply tissue migration of these cells. Moreover, absent in melanoma (AIM)2, a DNA damage sensor important in initiating the senescent phenotype, is upregulated in inflamed arteries of GCA patients [91].

Another effect of aging becomes apparent when analyzing macrophage responses to TLR stimulation, which have been implicated in GCA pathogenesis as well. TLRs are pathogen recognition receptors (PRRs) that enable macrophages to detect PAMPs, produce pro-inflammatory cytokines and trigger inflammation. Studies on peripheral blood monocytes from young and elderly participants showed that TLR4 expression was elevated in elderly participants in response to TLR stimulation and associated with elevated IL-8 levels [92]. TLR gene expression analysis in six different human vessel types showed vessel-specific expression of TLRs 1 to 9. TLR2 and TLR4 were abundantly expressed in medium- and large-vessels, while TLR7 and TLR9 were detected at low levels [93,94]. Interestingly, Rodriguez et al. investigated the expression and function of TLRs in GCA patients and revealed an increased TLR7 expression on circulating monocytes from GCA patients with active disease compared to healthy controls. However, despite this higher expression of TLR7, patient monocytes responded with a dampened pro-inflammatory cytokine secretion after stimulation with TLR7 agonists [95]. Investigating possible factors that could affect the TLR7 response showed that neither genetic defects nor amino acid substitutions in TLR7 were responsible for the observed effects. More-in depth research is needed to identify the cause of decreased TLR7 responses, whether due to inflammatory processes or overstimulation by ligands such as single-stranded RNA viruses.

### 1.6. Macrophage Related Biomarkers as a Tool for GCA Diagnosis and Disease Monitoring

Although the gold standard for GCA diagnosis is a TAB with evidence of vascular inflammation, the invasiveness of the procedure, the lack of sensitivity, as well as the risk of false-negative assessments due to patchy inflammation hampers the utility of a TAB as a diagnostic tool [96]. Recently, imaging techniques such as ultrasonography, detecting the “halo” sign in inflamed cranial and axillary arteries of GCA patients, and ^18^F-Fluorodeoxyglucose PET/CT (FDG-PET/CT) are emerging as more sensitive and specific diagnostic tools for GCA [97,98,99]. Although vasculitic lesions as detected by these imaging methods often improve upon treatment-induced remission, vascular abnormalities may still persist despite clinical remission [100,101]. This observation could reflect ongoing smoldering inflammation in the vessel or perhaps post-inflammatory vascular remodeling. Besides imaging techniques, current blood markers for GCA diagnosis are not disease-specific, have low diagnostic accuracy and cannot predict a non-favorable disease course. Therefore, more specific macrophage-targeted imaging tools and novel macrophage-derived serum markers may potentially be exploited to improve diagnostic accuracy and monitoring of disease activity in GCA patients, including patients on GC treatment [102].

#### 1.6.1. A. Serum Markers

GCA is a heterogeneous disease, and the extent of the local and systemic inflammatory response may differ among patients [103]. Early detection is important due to the danger of serious ischemic events, leading to blindness or stroke, requiring timely start of treatment. So far, several non-specific biomarkers (CRP, ESR, Serum amyloid A (SAA), IL-6) have been identified that can help to distinguish GCA from healthy controls [102]. However, the identification of new biomarkers to further characterize patient heterogeneity (disease subsets) can help to implement improved personalized medications for GCA patients [103].

**Figure 3:**
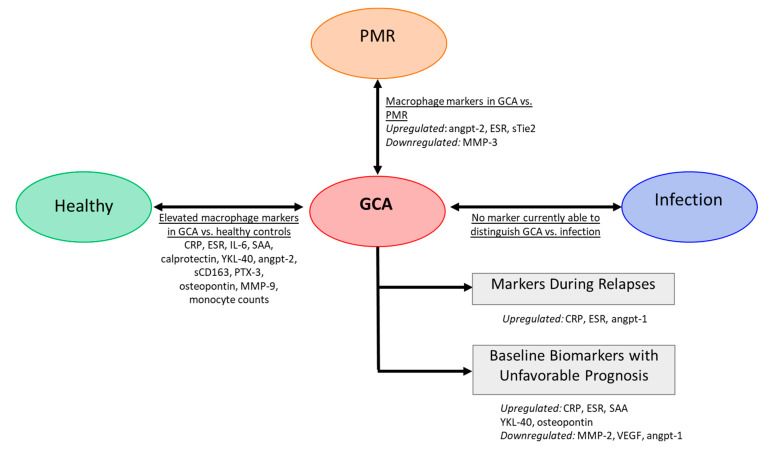
**Serum markers in GCA.** Several markers are altered in GCA compared to healthy controls. Some studies reported markers to differentiate GCA patients from patients with isolated polymyalgia rheumatica (PMR). There is currently no macrophage markers to discriminate between GCA and infection (e.g., respiratory and urinary infection) [51,104,105,106,107,108,109,110,111,112].

As novel biomarker candidates, proteins released by macrophages important in the pathology of GCA may provide a source of biomarkers for diagnosis and tracking disease activity. Indeed, levels of macrophage products such as soluble CD163, YKL-40, Angiopoietin-2 (Angpt-2), IL-33, VEGF, MMP-9, calprotectin and osteopontin were found elevated in the serum of GCA patients compared to healthy donors [51,104,105,106,107,108,109,110,111,112] (Figure 3).

Ideally, a biomarker or combination of biomarkers should be disease-specific. Investigations on biomarkers to distinguish concurrent arterial inflammation in patients phenotypically presenting as PMR demonstrated that high levels of angiopoietin-2, ESR and low MMP-3 levels at baseline may aid the diagnosis of concurrent vasculitis in PMR [113,114]. Although differences in macrophage-related markers may potentially distinguish GCA patients from patients with isolated PMR, distinguishing GCA patients from patients with infections is still a challenge, as high-level acute phase protein (CRP) is also elevated in patients with active infection. Infection triggers an acute inflammatory response, whereas GCA leads to more chronic granulomatous inflammation. As different inflammatory microenvironment may skew macrophages into different subtypes, macrophage products may outperform CRP, thereby improving the diagnostic process. However, such markers have yet to be identified (Figure 3).

Markers reflecting monocyte/macrophage activity may also serve as prognostic biomarkers. High levels of VEGF and Angiopoietin-1 (Angpt-1) and low levels of YKL-40 at baseline predicted a short time to GC-free remission in GCA patients and thus may identify easy to treat patients [104]. Serum levels of osteopontin may also have prognostic value as they were found to predict a relapsing disease course [110,111]. Additionally, MMP-2 was found to be negatively associated with relapse in GCA patients, while SAA, CRP, ESR were positively associated [102]. Other studies, however, failed to confirm the prognostic value of acute-phase markers CRP, ESR and SAA [104,115] (Figure 3).

Lastly, in addition to their utility in the diagnosis and prognosis of GCA, several serum biomarkers are gaining importance in monitoring disease activity. Currently, CRP, ESR and other inflammatory markers are used for monitoring patients. However, the specificity/sensitivity of CRP and ESR in predicting relapses in GCA was shown to be poor [17,116] (Figure 3). Although GC-sparing agents are developed, CRP and ESR remain unreliable as prediction markers for GCA flares. Recently, combined treatment with tocilizumab (IL-6R blockade) and GCs was shown to be beneficial for GCA patients. However, in tocilizumab-treated patients CRP levels and ESR, which are highly dependent on IL-6, are completely suppressed making it even more difficult for physicians to monitor disease activity [116]. Therefore, other markers that can flag relapses are needed and macrophage-derived markers might be good candidates. There is evidence that GCA symptoms can reappear as a result of smoldering inflammation in the tissue, despite suppression of systemic inflammation. Ultrasound imaging showed that vessel-wall thickening continues in GC-treated GCA patients [11]. In follow-up, temporal artery biopsies in GCA patients showed ongoing vascular inflammation with macrophages and T cells [15], which underscores the potential of macrophages or macrophage-derived factors in disease monitoring. As an example, our group recently demonstrated that serum levels of two macrophage-derived proteins, Calprotectin and YKL-40, remained elevated in GCA patients during the first year of treatment. It is tempting to speculate that the persistence of elevated calprotectin and YKL-40 levels reflects persisting vessel wall inflammation although this was not formally proven [104].

#### 1.6.2. B. Macrophage Targeted Imaging

Apart from blood biomarkers, positron emission tomography combined with computed tomography (PET/CT) is emerging as a potent diagnostic tool for GCA. Visualization of inflammation via [^18^F]FDG-PET imaging is now a useful tool for diagnosis and monitoring of treatment in GCA [101]. However, [^18^F]FDG-PET imaging still shows a number of disadvantages. Firstly, the diagnostic utility of [^18^F]FDG-PET in glucocorticoid-treated patients is significantly reduced only after 10 days of glucocorticoid treatment [117,118]. Yet, it is not always feasible to perform diagnostic and disease monitoring imaging of the patients within the narrow timeframe due to limited hospital capacities. [^18^F]FDG-PET scan relies on glucose uptake of metabolically active immune cells and stromal cells. Treatment with GCs was shown to reduce glycolysis in immune cells and therefore may downmodulate the vascular wall uptake of FGD [101]. Higher [^18^F]FDG uptake in aging vessels due to changes in metabolic activity, persistent vessel wall remodeling, and atherosclerotic calcifications may also pose a problem in the diagnosis of an aging disease such as GCA [119,120]. Therefore, recently discovered radiotracers for visualizing specific macrophage subsets may improve the diagnosis as well as the monitoring of treatment efficacy and disease progression during follow-up [121,122].

The fact that macrophages play key roles in various inflammatory diseases has encouraged many researchers to extensively explore the viability of targeting macrophages for diagnostic purposes. Several of these radiotracers are still in preclinical development but some radiotracers are already being studied in clinical trials (Table 1). One of the potential macrophage targeted radiotracers for the diagnosis and treatment monitoring of GCA is [^18^F]NOS, a radiopharmaceutical targeting iNOS. [^18^F]NOS was utilized in a study to quantify iNOS expression from endotoxin-induced lung inflammation in healthy volunteers. In this study, they showed that imaging iNOS activity is efficient in acute lung inflammation with abundant iNOS+ macrophage infiltration [123]. Considering the expression of iNOS in metabolically active macrophages and the presence of CD68(+)iNOS(+) macrophages in inflamed arteries of GCA patients, iNOS could be a candidate tracer to image macrophages in GCA.

Apart from iNOS+ macrophages, our recent observations highlighting the presence of CD206+ and FRβ+ macrophages in GCA have sparked interest in exploring novel radiotracers specifically targeting these macrophage subsets in GCA. In a first clinical study, [^18^F]fluoro-PEG-folate, a novel radiotracer that targets the FRβ was recently shown to be a potent tool in imaging FRβ macrophages at the site of inflammation in rheumatoid arthritis [124]. More recently, a clinical grade gallium-68-tagged antibody fragment targeting CD206 ([^68^Ga]NOTA-anti-MMR-sdAb) has been developed for phase I clinical studies in humans [136]. In addition, a ligand for the translocator protein (TSPO) on activated macrophages, 11C-(R)-PK11195, was shown to improve imaging of macrophage infiltration to vessel wall in large- vessel vasculitis patients [137]. Apart from the aforementioned radiotracers, several other radiotracers currently in preclinical development may also potentially be useful for disease monitoring in GCA. Targeting CD163 by ^68^Ga labeled anti-CD163-antibody in rats with acute collagen-induced arthritis (CIA) displayed a significant uptake at the site of inflammation [129]. Likewise, MMP-2/9 targeting [^68^Ga]DOTA-TCTP-1 showed a specific uptake in inflamed atherosclerotic lesions in mice [130]. To conclude, a variety of macrophage targeted imaging tracers are currently being developed and evaluated. These novel radiotracers may potentially be useful for diagnostic and disease monitoring purposes in GCA patients. 

### 1.7. Targeting Macrophages as Alternative Therapeutic Strategies for the Treatment of GCA

Glucocorticoids are currently the cornerstone in the treatment of GCA. GC treatment may resolve systemic inflammation rapidly but is not able to completely suppress vascular inflammation, leading to a relapsing/chronic disease. Moreover, long-term high doses of GCs come with serious adverse events in GCA patients [13,138,139]. Therefore, identification of GC-sparing agents for GCA treatment is imperative. Studies on temporal arteries engrafted into SCID mice demonstrated that after administration of dexamethasone for one week, T cells and macrophage functions were partially suppressed via blockage of the nuclear localization of NFkappaB which markedly reduced IL-6, IL-2, IL-1β and iNOS mRNA levels [140]. Chronic steroid therapy was shown to deplete some T cell products such as IL-17 but not IFN-γ, whereas, in macrophages, TGF-β1 synthesis was not affected [140,141]. This implies that T-cells and macrophages are only partially suppressed by GCs at the vascular site which may underlie the chronicity of the disease and thus emphasizes the need for alternative therapy in GCA. Studies revealed that increased CD68+ cells in TABs are linked to relapses with patients eventually requiring Disease-modifying antirheumatic drugs (DMARDs) [142]. Furthermore, persistence of macrophage infiltration in the vessels of GCA patients while on treatment, indicates that current treatments do not sufficiently suppress the local inflammatory response [15]. Therefore, targeting macrophages or macrophage-related pathways may prove to be more effective for suppressing chronic ongoing vascular inflammation and may provide lasting remission.

Indeed, IL-6 signaling, a cytokine released also by macrophages at the inflammatory site, has an essential role in systemic inflammation in GCA, as its serum levels correlate with CRP and other acute-phase proteins [19]. In the Giant-Cell Arteritis Actemra (GiACTA) trial, blocking the IL-6 receptor with tocilizumab was found to induce higher rates of sustained GC-free remission in GCA patients compared to placebo [19]. However, despite the clear efficacy of tocilizumab in GCA, up to 44% of patients treated with tocilizumab did not achieve sustained GC-free remission after 1 year of treatment. This underlines the heterogeneity among patients with GCA and the need for other targets for more personalized GCA treatment [19]. Furthermore, there is ongoing debate over the efficacy of tocilizumab in resolving vascular inflammation in GCA despite normalized inflammatory markers [143,144,145]. Additional to targeting IL-6 receptor with tocilizumab, sirukumab (IL-6 neutralization) and inhibition of the Janus kinase–signal transducers and activators of transcription (JAK–STAT) pathway [2] are being investigated as possible treatment options to resolve inflammation in GCA patients. The JAK-STAT pathway acts downstream of IL-6, IFN-γ and GM-CSF [146,147]. Therefore, targeting IL-6, IFNγ, GM-CSF or downstream cytokine signal transduction pathways may provide efficient treatment options in GCA.

Besides the IL-6 signaling pathway, the GM-CSF signaling pathway may be an important target for treatment in GCA. GM-CSF transcripts and protein were detected in vascular lesions and reported to play a major role in the pathogenesis of GCA [44,148]. In line with these reports, Mavrilimumab, a GM-CSF receptor antagonist is currently being evaluated as a therapeutic option for GCA patients. The phase 2 trial (NCT03827018) has demonstrated that 83% of Mavrilimumab treated patients were in sustained remission at week 26 compared to 50% of placebo-treated patients, when added to a 26-week prednisolone taper [149,150].

## 2. Future Perspectives

Investigating macrophage phenotypes and functions in GCA pathogenesis may facilitate the development of novel therapeutics and monitoring tools. Apart from intervening in cytokine-related pathways, targeting metabolic activity in macrophages may be an interesting alternative. Targeting glucose metabolism with anti-diabetic drugs in three PMR patients with concurrent type II diabetes improved the PMR symptoms and laboratory measurements like CRP [151]. Furthermore, small molecules such as DASA-58, TEPP-46, or shikonin, which target glucose metabolism led to a shift in macrophage phenotype by suppression of pro-inflammatory cytokine production in vitro [152,153]. Thus, targeting the glucose metabolism in GCA with small molecules may provide a novel option for GCA treatment. Clearly, this remains to be investigated in dedicated clinical trials. Lastly, considering the involvement of senescent cells in the production of pro-inflammatory mediators, ablation of senescent cells by senolytics is another therapeutically interesting concept. However, to be considered as a possible targeted therapy in GCA, the senescent cells contributing to the SASP in affected tissues needs first to be investigated [154].

## 3. Conclusions

In this review, we underlined the central role of monocytes and macrophages in the pathogenesis of GCA. In inflamed arteries, infiltrated and differentiated macrophages are key in shaping cellular and molecular processes involved in tissue destruction and tissue remodeling. Reversely, different cell types and their products such as metabolites, cytokines and chemokines in the vascular microenvironment influence differentiation, function, and heterogeneity of macrophages in GCA. Further research into the role of these constantly changing macrophages and their products in vasculitis lesions in GCA may eventually lead to discovery of new relevant targets for diagnosis, prognosis, monitoring disease activity, imaging and therapeutic intervention in GCA.

## Figures and Tables

**Table 1:** Radiotracers targeting macrophage subsets in the GCA affected vessel-wall.

Tracer Target Defined in GCA	References	Radiotracers	Status	References
iNOS	[53]	[^18^F]NOS	Clinical	[123]
FRβ	[44]	[^18^F]PEG-folate	Clinical	[124]
		3′-aza-2′-[^18^F]fluorofolicacid	Preclinical	[125]
		[^68^Ga]NOTA-folate	Preclinical	[126]
		[^18^F]AzaFol-based PET/CT	Preclinical	[127]
		[^18^F]FOL	Preclinical	[128]
CD163	[104]	[^68^Ga]anti-CD163-antibody	Preclinical	[129]
MMP2/MM9	[58,59]	[^68^Ga]DOTA-TCTP-1	Preclinical	[130]
		[^68^Ga]NOTA-C6	Preclinical	[131]
CD206	[44]	[^18^F]FDM	Preclinical	[132]
		[^68^Ga]NOTA-MSA	Preclinical	[133]
		[^18^F]FB-anti-MMR	Preclinical	[134]
		[^64^Cu]MAN-LIPs	Preclinical	[135]
		[^68^Ga]NOTA-anti-MMR-sdAb	Clinical	[136]

## Data Availability

Not applicable.
